# Recommendations for sample pooling on the Cepheid GeneXpert^®^ system using the Cepheid Xpert^®^ Xpress SARS-CoV-2 assay

**DOI:** 10.1371/journal.pone.0241959

**Published:** 2020-11-09

**Authors:** Michael G. Becker, Tracy Taylor, Sandra Kiazyk, Dana R. Cabiles, Adrienne F. A. Meyers, Paul A. Sandstrom

**Affiliations:** 1 National HIV and Retrovirology Laboratory, National Microbiology Laboratory, JC Wilt Infectious Diseases Research Centre, Public Health Agency of Canada, Winnipeg, Canada; 2 Department of Medical Microbiology and Infectious Diseases, University of Manitoba, Winnipeg, Canada; "INSERM", FRANCE

## Abstract

The coronavirus disease 2019 (Covid-19) pandemic, caused by SARS-CoV-2, has resulted in a global testing supply shortage. In response, pooled testing has emerged as a promising strategy that can immediately increase testing capacity. In pooled sample testing, multiple samples are combined (or pooled) together and tested as a single unit. If the pool is positive, the individual samples can then be individually tested to identify the positive case(s). Here, we provide support for the adoption of sample pooling with the point-of-care Cepheid Xpert^®^ Xpress SARS-CoV-2 molecular assay. Corroborating previous findings, the limit of detection of this assay was comparable to laboratory-developed reverse-transcription quantitative PCR SARS-CoV-2 tests, with observed detection below 100 copies/mL. The Xpert^®^ Xpress assay detected SARS-CoV-2 after samples with minimum viral loads of 461 copies/mL were pooled in groups of six. Based on these data, we recommend the adoption of pooled testing with the Xpert^®^ Xpress SARS-CoV-2 assay where warranted based on public health needs. The suggested number of samples per pool, or the pooling depth, is unique for each point-of-care testing site and can be determined by the positive test rates. To statistically determine appropriate pooling depth, we have calculated the pooling efficiency for numerous combinations of pool sizes and test rates. This information is included as a supplemental dataset that we encourage public health authorities to use as a guide to make recommendations that will maximize testing capacity and resource conservation.

## Introduction

The coronavirus disease 2019 (COVID-19) pandemic has caused an unprecedented demand for global testing supplies. In response, public health officials are searching for innovative ways to increase testing capacity in the face of limited resources. One approach that could be rapidly deployed to increase SARS-CoV-2 testing capacity is pooled sample testing, a method that involves mixing multiple samples and testing them as a single unit, thereby decreasing the resources required to test multiple samples [1–3]. The number of samples that may be pooled, i.e. the pooling depth, is determined by the sensitivity of the test method as well as the prevalence of disease within the community. Recently, laboratories have reported success when pooling 10 [1], 30 [2], and 48 [3] samples when using the Corman quantitative reverse transcription PCR (RT-qPCR) test [4]. Similar strategies should also be explored for the SARS-CoV-2 point-of-care tests, such as the Cepheid *Xpert*^®^ *Xpress SARS-CoV-2 assay*.

The Cepheid *Xpert*^®^ *Xpress SARS-CoV-2 assay* was approved by Health Canada on March 24^th^, 2020 under interim order authorization and is a rapid, fully-automated, and self-contained multiplex qualitative RT-qPCR test for SARS-CoV-2 detection that uses single-use cartridges and has a run time of 50 minutes. This assay targets two regions of the SARS-CoV-2 genome: the nucleocapsid (N) and the envelope (E) regions and is deemed positive when either of the two regions amplify before cycle 45 (i.e. produce a fluorescent signal or crossing threshold [Ct] <45). Evaluation of the Cepheid SARS-CoV-2 assay is ongoing, however, it has shown increased sensitivity compared to the Abbott *ID Now SARS-CoV-2 Assay* [5, 6], and has high agreement (>99%) with the Roche Cobas 6800 system [5, 7, 8] and the Centers for Disease Control and Prevention (CDC) RT-qPCR test [8]. Using viral recombinant samples, Cepheid reports 100% sensitivity (n = 35) at 250 copies (cp)/mL whereas with synthetic RNA controls (that do not consider signal loss during nucleic acid extraction), Zhen et al. [6] reported 100% sensitivity at 100 cp/mL (n = 10) and 87.5% sensitivity at 50 cp/mL (n = 8).

Given the high sensitivity of the *Xpert*^®^ *Xpress SARS-CoV-2 assay*, it is reasonable to propose that this method could be suitable for pooled sample testing. Here, the potential for pooled SARS-CoV-2 testing was assessed on the GeneXpert system using a small panel of clinical specimens which had low- to mid-range viral loads that were diluted with known clinical negative samples. The results herein corroborate previous findings that the limit of detection for the Cepheid assay is likely <100 cp/mL. Additionally, data generated by this study suggest that the GeneXpert device can be effective for detecting SARS-CoV-2 in pools containing six individual samples. Finally, a reference dataset is provided that can be used by public health authorities to advise point-of-care sites on the optimal number of samples that can be pooled given their current positive test rates.

As the *Xpert*^®^ *Xpress SARS-CoV-2 assay* is a rapid near-point-of-care assay, samples are generally processed as they become available. An added advantage of the system is that it can be operated by users with a limited background in molecular biology; however, these users may not be comfortable processing large numbers of samples simultaneously. Because of these factors, we did not investigate large (>10) pool sizes on the GeneXpert, although they are theoretically possible with this system. We generally recommend six samples or less within pools at sites employing the GeneXpert system.

## Materials and methods

### Viral culture

High-titre SARS-CoV-2 culture (Strain VIDO; GISAID Accession: EPI_ISL_425177), made inactive by gamma-irradiation, was provided by the Special Pathogens Program of the National Microbiology Laboratory. Briefly, the virus was cultured in Vero cells in minimum essential media, and cellular debris was removed via low-speed centrifugation. Serial dilutions of the high-titre SARS-CoV-2 culture were tested on the GeneXpert. The GeneXpert returns cycle threshold (Ct) values, or the number of PCR cycles needed before a signal can be detected. The Ct value decreases as sample input increases, and can be used for quantification. With this method, viral load of the inactivated virus was determined by comparing the Ct values to a standard curve that was formed using serial dilutions of recombinant Sindbis virus-containing SARS-CoV-2 RNA from the *SeraCare AccuPlex™ SARS-CoV-2 Reference Material Kit* (0505–0126).

### Clinical specimens

Clinical nasopharyngeal swab samples which were collected in 1 mL of viral transport media were provided by Cadham Provincial Laboratory (CPL; Winnipeg, Canada). All samples were previously characterized by CPL using an approved SARS-CoV-2 diagnostic RT-qPCR assay. The panel used for this study consisted of six positive CPL clinical samples plus an additional two low viral load swab samples (Ct = 37/Ct = 38), which were provided by the Influenza and Respiratory Viruses Program at the National Microbiology Laboratory. Pooled negative samples were also included and were provided by the Influenza and Respiratory Viruses Program. All samples used for this study were ethics-exempt, anonymized, remnant diagnostic samples. The authors were not involved in sample collection, and the samples were not collected specifically for this study.

### Standard curve for the Xpert Xpress^®^ SARS-CoV-2 assay

A standard curve was used to quantify the SARS-CoV-2 viral load in each of the clinical samples used in this study. To produce the curve, 10-fold serial dilutions of inactivated high-titre SARS-CoV-2 were prepared in viral transport media to yield a series ranging from 6 x 10^8^ cp/mL to 6 x 10^0^ cp/mL. For each dilution, 300 μL was pipetted into an Xpert^®^ Xpress SARS-CoV-2 cartridge. The standard curve linear equation for the E and N amplicons were then used to determine the viral load of the clinical samples. The reported viral load is the average between the N and E targets.

### Sample pool tests

All testing and standard curve preparation was performed on the same GeneXpert system using the same module. Initially, each sample was tested without pooling and Ct values were used to determine the viral load. Each sample was then diluted in a pool of confirmed negative clinical specimens to simulate either three- and six-sample pools (corresponding to a three-and six-fold dilution, respectively). Due to a shortage of assay cartridges, only the six-sample pools were performed in triplicate and a limited number of samples were included in our panel. Each replicate pool was created independently.

### Calculation of pooling efficiency

To guide efficiency, the impact of pooling on testing capacity was calculated at various pooling depths (1–10) and test positivity rates (0–100%). Similar to the statistical approach used by Hanel and Thurner [9], a custom Python script was used to calculate the pooled testing capacity relative to non-pooled testing capacity, represented as a percentage, for each combination. A value greater than 100% indicates that testing capacity has increased, whereas values below 100% indicate decreased capacity. To calculate the relative testing capacity of the pool sizes and positive test rates, the proportion of pools that were SARS-CoV-2 positive (*P*^*S+*^) were determined with the following equation:

$$P^{S+}=1-{\left(1-p\right)}^{n}$$

where *n* is the pool size and *p* is the proportion of individual tests that are positive. The average number of tests needed per pool was then determined by multiplying the proportion of positive pools by their size, which indicates how many tests are required to resolve the SARS-CoV-2 status of individual samples within positive pools. In addition, this equation considers the original test that was needed for the pool itself (+1). The average number of tests (*T*) needed to process each pool is therefore determined by:

$$T={(P}^{S+}x\;n)+1$$

Finally, relative testing capacity was calculated by dividing the average number of tests required for each pool, divided by the number of samples tested:

$$Relative\;Testing\;Capacity=\frac{T}{n}x\;100\%$$

## Results

### Xpert^®^ Xpress SARS-CoV-2 assay for quantitation

**Fig 1:**
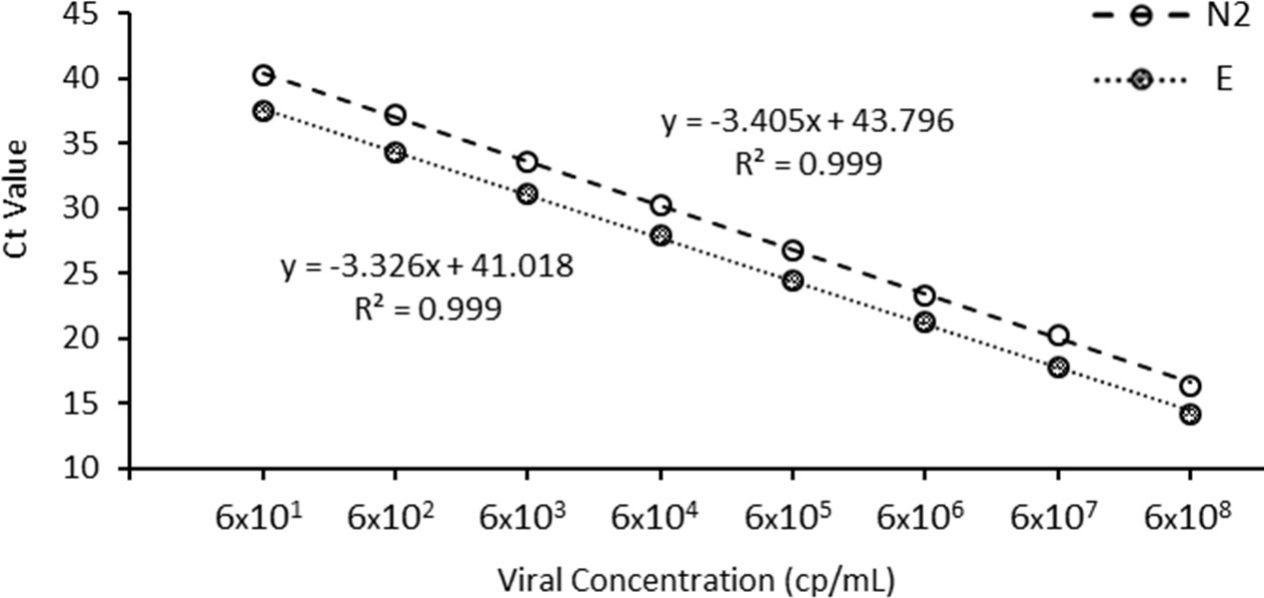
Standard curve for the *Xpert*^*®*^ *Xpress SARS-CoV-2 assay*. Assay targets both the nucleocapsid (N; empty circle with a dashed line) and envelope (E; filled circle with a dotted line). The curve was produced using serially-diluted gamma-irradiated virus culture (GISAID Accession: EPI_ISL_425177) produced at the National Microbiology Laboratory.

Although the Xpert^®^ Xpress SARS-CoV-2 assay is considered to be a qualitative test, it can approximate viral loads through the use of Ct values (the number of qPCR cycles needed to reach detection) and a standard curve. All dilutions above 60 cp/mL were recorded as SARS-CoV-2 positive by the assay (S1 Table), consistent with the previously observed limit of detection [6] for the Xpert^®^ Xpress SARS-CoV-2 assay of <100 cp/mL. The resulting curve was highly linear (R^2^ > 0.999), suggesting that the Ct values can be used for quantitation (Fig 1).

### Sample pooling and sensitivity

To determine the effect of sample pooling on the sensitivity of the Xpert^®^ Xpress SARS-CoV-2 assay, five clinical samples were selected with Ct values ranging from 23–35 as determined by the Corman RT-qPCR test performed at the CPL in Winnipeg, MB. Each sample was tested individually prior to pooling and the resultant Ct values were converted to viral load using the standard curve. Input viral loads ranged from approximately 938 cp/mL to 2.85 million cp/mL (Table 1). Each of these samples were then diluted into three or six sample pools using SARS-CoV-2 negative clinical samples. Although Ct values were higher following pooling, the Xpert^®^ Xpress SARS-CoV-2 assay was able to correctly identify the SARS-CoV-2 positive pools. Standard deviation of Ct values between replicates increased with increasing Ct values, likely due to sampling and PCR biases.

**Table 1:** Five clinical samples collected at the Cadham Provincial Laboratory (CPL) were selected for analysis with Ct values ranging from 23–35 as determined by the CPL in-house RT-qPCR test.

Sample ID	RT-qPCR Ct Value	Nominal Viral Load (cp/mL)	Undiluted			Three Sample Pool			Six Sample Pool (Replicate 1)			Six Sample Pool (Replicate 2)			Six Sample Pool (Replicate 3)			Replicate Standard Dev.		
			*E*	*N2*	*SPC*	*E*	*N2*	*SPC*	*E*	*N2*	*SPC*	*E*	*N2*	*SPC*	*E*	*N2*	*SPC*	*E*	*N2*	*SPC*
CPL1	23	2,452,553	22.2	24.7	29.2	22.8	24.3	24.3	24.3	26.2	27.3	23.8	25.8	27.6	24.3	26.5	27.3	0.21	0.29	0.14
CPL2	26	154,663	26.1	28.9	28.7	27.1	28.0	28.0	28.0	30.6	27.8	28.2	30.9	28.1	28.0	30.8	27.7	0.09	0.12	0.17
CPL3	31	6439	30.5	33.9	28.4	31.9	33.5	33.5	33.5	37.4	28.1	34.1	36.7	27.9	33.5	36.6	28.2	0.37	0.36	0.12
CPL4	33	2245	32.0	35.5	28.6	33.3	35.6	35.6	35.6	37.7	27.4	33.0	36.1	27.7	35.6	38.7	27.7	1.07	1.07	0.14
CPL5	35	938	33.6	36.3	28.0	36.2	40.7	40.7	40.7	41.1	27.6	39.0	39.2	27.6	40.7	39.1	27.7	1.77	0.92	0.05

Each sample was tested with the *Xpert*^*®*^ *Xpress SARS-CoV-2 assay* as an undiluted sample, or diluted three or six-fold in negative clinical samples to simulate a three or six (performed in triplicate) sample pool. Ct values are provided for the envelope (E), nucleocapsid (N), and sample processing control (SPC) targets at each dilution. Nominal viral load of the clinical samples was determined using a standard curve of the *Xpert*^*®*^ *Xpress SARS-CoV-2 assay*. For the six sample pool replicates, standard deviation was calculated for each target.

To better observe the effects of sample pooling near the assay’s limit of detection, an additional three clinical samples that had high Ct values (>37) were selected. This included a discordant sample that was not detected by the CPL RT-qPCR test but was subsequently identified as weakly positive on the GeneXpert (CT = 43.5/39.2). At initial viral loads of 461 and 1362 cp/mL, the Xpert^®^ Xpress SARS-CoV-2 assay detected SARS-CoV-2 after six-fold pooling, while the weak positive (64 cp/mL) returned a negative result (Table 2). Additionally, the E target was not detected in one of the pools; however, only one detected analyte is needed to be considered positive.

**Table 2:** An additional three clinical specimens with high Ct values were selected to observe the effect of sample pooling close to the limit of detection of the *Xpert*^*®*^ *Xpress SARS-CoV-2 assay*. This included two samples provided by the National Microbiology Laboratory and one from the Cadham Provincial Laboratory (CPL), which is a discordant sample not detected by CPL’s Corman RT-qPCR test, but detected as a weak positive by the Xpert^®^ assay. At the six-fold dilution, the weak positive was no longer detected by the assay.

Sample ID	RT-qPCR Ct Value	Nominal Viral Load (cp/mL)	Undiluted			Six Sample Pool		
			*E*	*N2*	*SPC*	*E*	*N2*	*SPC*
NML1	37	1362	32.8	36.1	27.8	38.9	39.2	27.8
NML2	38	461	34.9	37.1	29.4	ND*	38.9	27.7
CPL6	ND*	64^†^	43.5	39.2	28.3	ND*	ND*	28.2

* ND; Not Detected

† Ct value was outside of the standard curve (E) and viral load was inferred through extrapolation

### Determining the optimal pool size

**Fig 2:**
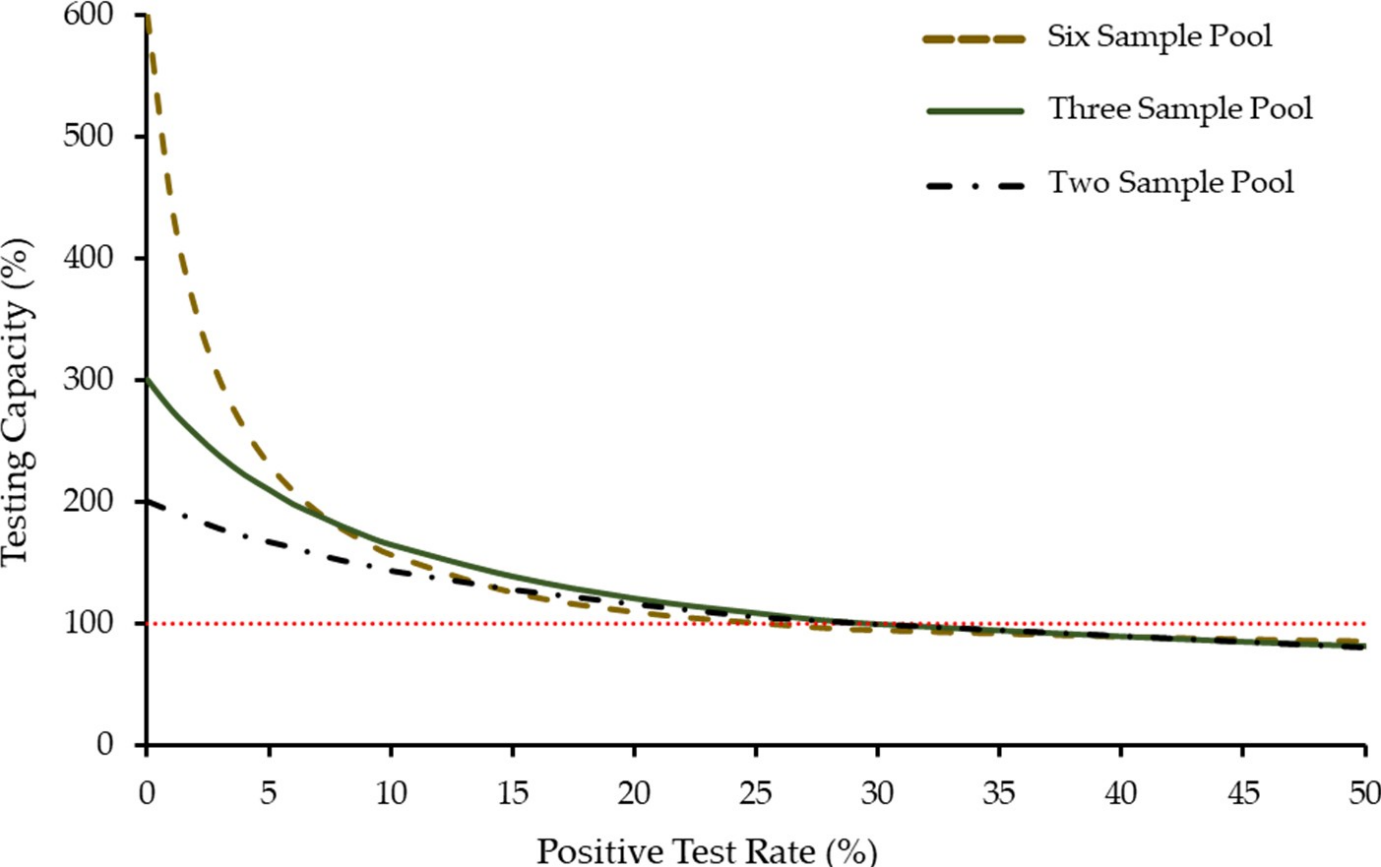
The effect of sample pooling on testing capacity at different pool sizes. Testing capacity is shown for pools of two (black dashed-dotted line), three (green solid line), or six (brown dashed line) samples. For each pool size, testing capacity is plotted against the rate of positive individual tests. The red dotted line represents the point at which pooled testing decreases capacity and is no longer viable. The cross-over point, when three sample pooling is more efficient than six sample pooling, occurs at 7.6%.

An objective of this study was to provide guidance for when sample pooling is a viable option for SARS-CoV-2 testing with the *Xpert*^*®*^ *Xpress SARS-CoV-2 assay* or any sensitive SARS-CoV-2 test in general. At high positive testing rates, pooling may increase the number of tests required to screen samples and increase turnaround time. Furthermore, deciding which pooling depth to use is arbitrary without understanding the relationship between pooling depths and positive test rates. We determined the testing capacity of various pool sizes (1–10) and test rates (0–100% in increments of 0.1%). A complete summary of all of the combinations can be found in S2 Table, and a graphical representation of a subset of these data is shown in Fig 2.

This information can help medical authorities provide informed recommendations on sample pooling. For example, no pooling strategy is effective when positive test rates exceed ~30%. Additionally, a pool sizes of two are never more efficient than three sample pooling (Fig 2). Although at low positive test rates larger sample pools are favored, this quickly changes when test rates increase above 1%. For example, if the positive test rate at a site is ~3% the ideal pool size would be six samples.

## Discussion

The results of this study strongly suggest that sample pooling is a viable option for SARS-CoV-2 testing using the *Xpert*^*®*^ *Xpress SARS-CoV-2 assay*. All samples tested positive after pooling, except for a high-Ct discordant positive that had a low viral load (64 cp/mL). At this level of sensitivity, pooled tests should detect SARS-CoV-2 in the vast majority of clinically-relevant cases. For instance, a study following 80 patients at different stages of infection detected average viral loads of >10^4^ from 1 day before to 7 days after disease onset, using sputum (n = 67), throat (n = 42), and nasal (n = 1) swabs [10], with the lowest observed viral load of 641 copies/mL. Another research group determined average viral loads to be >10^5^ at the onset of mild to moderate symptoms [11]. When testing asymptomatic individuals, results still show typical Ct values of 22–31 with the Corman RT-qPCR assay [12–14]. Although Ct values cannot be determined when testing a pooled sample; they will be determined subsequently when the contributing samples are individually tested, and may provide important information on patient infectivity. For example, patients with a Ct value above 34 are unable to be cultured, suggesting decreased transmissibility [15, 16].

One challenge that may prevent some point-of-care testing sites from adopting a pooled testing strategy is the lack of mechanical pipettes. The *Xpert*^*®*^ *Xpress SARS-CoV-2 assay* is provided with single-use transfer pipettes that dispense 300 μL of sample. With small pool sizes, multiple samples can be combined into a 5 mL specimen tube or 15 mL canonical tube and inverted to mix. Subsequently, 300 μL of this pool can then be transferred into a test cartridge. With this approach, pooled testing with the *Xpert*^*®*^ *Xpress SARS-CoV-2 assay* could be readily achieved in a resource-limited setting with the provision of additional 300 μL transfer pipettes.

To prevent accidental cross-contamination or pipetting errors, we recommend that sites using the GeneXpert limit pool sizes to six samples, especially in resource limited settings. Samples can be pooled as needed in batches of 2–6 samples; however, we do not recommend pooling when positive test rates increase above 15%. Above positive test rates of 15%, the benefits of sample pooling are negligible, and are not worth the added risk of cross-contamination and increased turnaround time.

Other strategies for SARS-CoV-2 pooled testing are also being investigated such as combinatorial testing, or matrix testing [3, 17]. For this approach, samples are combined into multiple pools, such that each sample is tested multiple times across multiple pools. The combination of SARS-CoV-2 positive pools can identify individual positives with limited retesting required. Although this strategy is promising, it works best for high-throughput laboratories processing batches of hundreds of samples using 96- or 384-well plates and RT-qPCR machines. Because of the need for larger batch sizes and the more complicated testing design, a combinatorial approach is unlikely to be feasible with point-of-care tests which perform only a few tests in a single run, such as the *Xpert*^*®*^ *Xpress SARS-CoV-2 assay*.

Another pooling strategy proposed by the German Red Cross Blood Donor Service and Geothe University is swab pooling, or the mini-pool method [18]. Multiple swabs can be combined into a single tube at the point of collection, rather than the traditional method of pooling transport media or extracted RNA. As a result, there is minimal loss of sensitivity as no dilution is occurring. The main disadvantage of this approach is that the pooled samples need to be collected simultaneously at the same location, however, this approach may be applied in certain scenarios such as door-to-door household testing, workplace screening, or for blood donor screening. This approach could easily be combined with traditional pooling to substantially increase testing capacity with the *Xpert*^*®*^ *Xpress SARS-CoV-2 assay* or other validated molecular methods.

## Conclusions

This study serves as a resource that can be used to determine appropriate pool sizes for each testing site. Public health authorities can approximate positive tests rates, and use this information with the reference table (S2 Table) to make appropriate recommendations on pooling strategies. The application of sample pooling, when possible, can be used to immediately increase testing capacity on the GeneXpert^®^ system while conserving resources. Future experiments should investigate if more extensive pooling is viable on the GeneXpert^®^ system, similar to the aggressive pooling strategies being explored for the laboratory-based RT-qPCR tests.

## Supporting information

S1 Table Serial dilutions of high-titre irradiated SARS-CoV-2 tested with the Xpert Xpress SARS-CoV-2 assay. (DOCX)

S2 Table Pooling efficiency grid for SARS-CoV-2 sample pooling with the GeneXpert Xpert Xpress SARS-CoV-2 assay. (XLSX)
