# Supplementary material for: Recommendations for sample pooling on the Cepheid GeneXpert® system using the Cepheid Xpert® Xpress SARS-CoV-2 assay
Source: PLoS One. 2020 Nov 9;15(11):e0241959. doi: 10.1371/journal.pone.0241959 (PMC7652318; doi:10.1371/journal.pone.0241959)
Supplement: S1 Table — (DOCX) [file pone.0241959.s001.docx]

**S1 Table.** Serial dilutions of high-titre irradiated SARS-CoV-2 tested with the *Xpert Xpress SARS-CoV-2 assay*. Results show Ct values of the envelope (E), nucleocapsid (N), and sample processing control (SPC) targets at each dilution.

| Approx. viral concentration (cp/mL) | Result | E | N | SPC |
| --- | --- | --- | --- | --- |
| 6 x 10^0^ | Negative | ND* | ND* | 28.3 |
| 6 x 10^1^ | Positive | 37.5 | 40.2 | 28.3 |
| 6 x 10^2^ | Positive | 34.3 | 37.2 | 27.9 |
| 6 x 10^3^ | Positive | 31.1 | 33.6 | 28.3 |
| 6 x 10^4^ | Positive | 27.9 | 30.2 | 28 |
| 6 x 10^5^ | Positive | 24.5 | 26.7 | 28.1 |
| 6 x 10^6^ | Positive | 21.2 | 23.3 | 27.5 |
| 6 x 10^7^ | Positive | 17.8 | 20.2 | 28.3 |
| 6 x 10^8^ | Positive | 14.1 | 16.4 | 27.8 |

*ND = Not Detected
